# The Treatment of Cancer by Arsenic

**Published:** 1900-06-16

**Authors:** 


### THE TREATMENT OF CANCER BY ARSENIC.

If one searches for the causes of those curious changes of fashion which from time to time seem to sway medical treatment in one direction or another, one sees that it is not altogether success or want of success which determines whether any particular form of treatment shall survive. Markedly this has been the case in regard to the employment of caustics in the treatment of malignant disease. In the days before anæsthetics and antiseptics, operations were apt to be limited in extent—far too limited to allow of that complete and radical removal of surrounding parts which we now know to be essential if a cure is to be obtained—and the lack of success which followed the partial operations then in vogue led many surgeons to cast about for something better than the knife. Naturally enough, in those days caustics were largely used. They did just what then the surgeon dared not do—they went wide of the disease; and just in proportion as they did so they succeeded. With the introduction of the modern "complete" operation, however, caustics have dropped quite out of fashion, and practically nothing has been heard of them for many years. This, however, was not entirely due to lack of success by the caustic method, but rather to the greatly increased facility with which extensive operations with the knife could be performed. For there was always something in caustics, and especially in such as contained arsenic, and men ahve not been wanting who maintained that by their local absorption these caustics exercised a more or less occult influence on cancer growths, which was not limited to the area in which they showed an absolutely destructive action. Still one has heard but little of this tretament for some time, but recently, however, Dr. Trunecek[1] has returned to this method, using a mixture of arsenious acid, one gramme to 75 grammes of absolute ethylic alcohol and the same quantity of distilled water. It is applied as follows: The ulcer is cleansed and dried, and, if necessary, scraped. The arsenical mixture is then stirred and spread over the whole surface of the tumour. It is not covered, but is left to dry; and if the patient feels no pain another layer may be spread over it. The day after, or the next, the neoplasm will be found to be covered with an eschar. This is not removed, but the applications are repeated daily. By degrees the crust thickens and invades the whole surface, and ultimately the whole thing falls out or becomes easily removable. As the eschar thickens the strength of the caustic may be increased. Ultimately, it is said, no trace of the cancerous tissue remains, and nothing is left but a granulating wound. We do not doubt it. All this was done often enough 40 years ago. The whole question is one of the comparative advantages of different methods. At present we cannot but feel that all evidence goes in favour of the knife. But do not let us be so prejudiced as to refuse to look at other methods. There is no doubt that the knife cures no further than it cuts, and the slightest error in judgment, the slightest timorousness which should hold back the operation from complete removal, might be fatal to success. The whole question, then, lies in this. Does arsenic exert a selective influence? Does it pick out and destroy cancer cells over a wider area than that over which its influence is destructive to all tissues alike? We do not think that Dr. Trunecek has proved this. His cases are good, but, then, many of them might have been cleared out by the knife just as well. Everything hinges on whether or no arsenic exercises a selective influence on cancerous tissue, and this, we think, is non-proven. Still, it is well that we should know what is being done. Dr. Trunecek summarises his idea as follows:—

1. The cancer cells are, first of all, dehydrated by the alcohol, and then their protoplasm coagulates in the presence of arsenic.
2. The cells of cancerous connective tissue—cancerous stroma—degenerate and provoke a serous exudation, which in its turn determines alterations in the cells mummified by the arsenic.
3. In the surrounding healthy tissues a circumscribed inflammation is set up, which always goes on to suppuration, by means of which the neoplasm is first cut off from the circulation, and finally eliminated as a foreign body.

[1] New York Medical Record, June 2.

